# Development of a multiplex PCR for detection of pathogenic Mycobacterium orygis in cattle tissues harboring tuberculous-like lesions

**DOI:** 10.21203/rs.3.rs-6947470/v1

**Published:** 2025-09-04

**Authors:** Sukhen Samanta, Premanshu Dandapat, Molla Zakirul Haque, Partha Sarathi Jana, Samiran Bandyopadhyay, Arnab Sen, Pramod Kumar Nanda, Subhasree Das, Keka Sarkar, Prasad Thomas, Abhishek V, Ayan Mukherjee, Sachinandan De

**Affiliations:** University of Kalyani; ICAR-Indian Veterinary Research Institute; West Bengal University of Animal and Fishery Sciences; West Bengal University of Animal and Fishery Sciences; ICAR-Indian Veterinary Research Institute, Eastern Regional Station; ICAR-Indian Veterinary Research Institute; ICAR-Indian Veterinary Research Institute, Eastern Regional Station; ICAR-Indian Veterinary Research Institute, Eastern Regional Station; University of Kalyani; ICAR-Indian Veterinary Research Institute; ICAR-Indian Veterinary Research Institute; West Bengal University of Animal and Fishery Sciences; ICAR-National Dairy Research Institute

**Keywords:** multiplex PCR, SNPs, Mycobacterium orygis, mbtG, fadD23

## Abstract

Mycobacterium orygis, a recently defined member species of Mycobacterium tubercuolsis complex (MTBC), is emerging as a major threat to zoonotic tuberculosis control, especially in the Asian Subcontinent. The dearth of low-cost diagnostic assay to differentiate M. orygis from other members of the MTBC leads to unavailability of information about the actual burden of this species in human and animal population. In this study, we developed a multiplex PCR for distinguishing M. orygis from other MTBC based on two M. orygis-specific nonsynonymous point mutations in mbtG and fadD23 genes identified by comparative genome analysis. The specificity of the assay shows that a 434 bp IS1081 fragment was amplified from common MTBC species including M. orygis while 240 bp and 181 bp mbtG and fadD23 gene fragments were amplified only from M. orygis. No amplification was observed for nontuberculous Mycobacterium (NTM) and non-Mycobacterial pathogens. The multiplex PCR assay showed a detection limit of 32 pg of M. orygis DNA. Furthermore, a total of 85 tuberculous-like lesions in the different tissues of slaughtered cattle were tested for identification of the M. orygis, and the results showed IS1081, mbtG and fadD23 amplicons in three tissue DNA extracts confirming they contain M. orygis DNA. Also, a single IS1081 amplicon was amplified from one tissue sample signifying presence of DNA of any MTBC species other than M. orygis. An established TaqMan real time PCR assay targeting region of differences (RD) in M. orygis genome was carried out to validate the result of the assay. This showed 100 % accuracy of the in-house developed multiplex PCR.

## Introduction

Tuberculosis (TB), an ancient disease with substantial impact on human civilization, till poses a serious threat to global health status with millions of cases identified each year and more than 1.6 million deaths (Global Tuberculosis Report, 2024, World Health Organization). The etiological agents of TB are genetically similar members of the *Mycobacterium tuberculosis* complex (MTBC) comprising of both animal- and human-adapted (*M. tuberculosis* sensu stricto) lineages. More than ten *Mycobacterium* species with substantially conserved genomes make up the MTBC (Bespiatykh et al. 2021). Zoonotic tuberculosis (zTB) is a type of TB which is transmitted between animal and human. An estimated 140000 cases of zTB occur each year resulting in approximately 11400 deaths worldwide (Global tuberculosis report 2020, World Health Organization). Cattle serve as the primary animal reservoir for TB concerning zoonotic transmission to humans; nevertheless, the illness can also impact various other species and establish itself within wildlife reservoirs. Although previously it was thought that *M. bovis* is the cause of zTB several other animal-adapted distinct MTBC lineages have now been confirmed as potential cause of the disease (Duffy et al. 2024).

*M. orygis*, formerly referred to as Oryx bacillus or the antelope clade, has been occasionally documented in relation to zTB throughout the previous three decades. With the rapid progress of genome sequencing and bioinformatic methods in the last few decades, the frequency of case reports of *M. orygis* infection has recently surged (Hugh et al. 2025). Despite the global context of the prevalence of *M. orygis* being insufficiently studied, specific geographical regions have significantly elevated case densities, especially in South Asia (Rufai et al. 2021). About 33.6% of total *M. orygis* cases have been reported from South Asian countries like Bangladesh, India, Pakistan, and Nepal (Hugh et al. 2025; Rani et al. 2025). 66% of *M. orygis* cases have been found in Canada, the Unites States of America, New Zealand, and the United Kingdom. Although these are low TB-burden countries [less than 10 per 100,000 cases of TB] but bovine TB is endemic in these countries (World TB incidence, Global TB Report, WHO, 2023). Recently, a slaughterhouse surveillance study conducted in our laboratory has identified two *M. orygis* isolates from slaughtered cattle in Kolkata, India (Haque et al. 2024). Nonetheless, zTB cases caused by *M. orygis* in animal populations have widespread impact on human health and food safety.

Despite progress in identifying and reporting *M. orygis* infections, the real burden of the zTB caused by *M. orygis* is still unknown. The challenge in distinguishing *M. orygis* from *M. tuberculosis* and other MTBC members using conventional methodologies has led to the insufficiency of information regarding the clinical manifestations of *M. orygis*, especially in situations where sub-speciation of MTBC is not customary (Soolingen et al. 1994; Lipworth et al. 2019). Species level differentiation of MTBC, including *M. orygis*, is a major challenge. A thorough genome mining and identification of potential genomic markers for MTBC lineage is necessary to develop specialized molecular diagnostic assays. Although MTBC members are genetically similar, each species has unique insertions and deletions known as region of differences (RD) (van Ingen et al. 2012). RD analysis shows presence of RD1 and RD4 and absence of RD7, RD8, RD9. RD analysis can also be used in tandem with SNPs to identify the species (Napier et al. 2020; Bespiatykh et al. 2021). Several SNPs have been identified which are exclusively present in *M. orygis* genome (Rani et al. 2025). All these SNPs have been identified by *in silico* analysis of sequenced genome of *M. orygis* isolated from diverse host ranges., RD10, and RD12 regions in *M. orygis* genome (van Ingen et al. 2012; Refaya et al. 2019). Two complete genome of *M. orygis* are there in NCBI database (Genbank accession no.: CP063804.2 and CP138660.1). Although several genomic markers like RDs and SNPs have been identified in *M. orygis* genome very few studies have used these structural variations for diagnosing *M. orygis* from biological samples by relatively simple and inexpensive method. Therefore, the present study was carried out to develop a fast and conventional PCR-based multiplex assay that can identify *M. orygis* from postmortem tissue samples accurately. Two nonsynonymous SNPs in *mbtG* and *fadD23* genes were used for developing the multiplex PCR. The *mbt* genes in mycobacteria plays role in the biosynthesis of mycobactin, a siderophore crucial for iron uptake and cellular survival of the bacteria. Fad23 is a protein involved in the synthesis of Sulfolipid-1, a vital constituent of Mycobacterial cell wall. We compared the genome of *M. orygis* from diverse host species and geographical areas and compared them with other MTBC genome. The unique SNPs found in *M*. *orygis* were identified and a multiplex PCR was developed to identify the two SNPs in *mbtG* and *fadD23* genes. Also, the in house developed PCR was validated with highly specific Taqman Real-time PCR assay.

## Materials and Methods

### 2.1 Bacterial strains and culture

*M. orygis*, other MTBC and NTM species were cultured in Lowenstein-Jensen (L-J) glycerol and L-J pyruvate solid media slants. All the isolates were previously isolated during earlier study (Haque et al. 2024), routine monitoring and subcultured in our laboratory in this study. We used six *M. orygis* strains confirmed by WGS analysis, two *M. tuberculosis* strains, one *M. bovis* strain (AN5), one *M. bovis* BCG strain for standardization of the assay. Details of the bacterial cultures used in the study are listed in Table 1.

### 2.2 Genome sequence analysis and primer design

The whole-genome sequences of *M. orygis* strain MUHC/MB/EPTB/Orygis/51145 (GenBank accession nos. NZ_CP063804.1), *M. orygis* strain NIAB_BDWBCSHFL_1 (GenBankaccession nos.NZ_CP138660.1), *M. tuberculosis* strain H37Rv (GenBank accession nos. NC_000962.3), *M. bovis* strain ATCC 35743 (GenBank accession nos. NZ_CP039850.1), *M. bovis* BCG strain Pasteur 1173P2 (GenBank accession nos NC_008769.1), *M. caprae* strain Algaeu (GenBank accession nos. NZ_CP016401.1), *M. microti* strain OV254 (GenBank accession nos. NZ_LR882499.1), *M. africanum* strain GM041182 (GenBank accession nos. NC_015758.1), *M. canetti* (GenBank accession nos: NC_015848.1) were downloaded from the NCBI genome database (https://ftp.ncbi.nlm.nih.gov/genomes). The comparative circular genome map of nine MTBC genome was built by BLAST Ring Image Generator (BRIG) (Alikhan et al. 2011). The comparative proteome map of the MTBC organisms was developed by BV-BRC proteome comparison tool (Olson et al. 2023). The SNPs unique in coding sequences of *M. orygis* are screened by snippytools of galaxy which finds SNPs between a haploid reference genome and compiled by snippycore (Seemann, 2015). Out of identified SNPs two non-synonymous SNPs were randomly selected in two genes *long-chain-fatty-acid--CoA ligase FadD23* (*fadD23*, T>G transversion at 218 position in *M. orygis* resulting in Leu→Arg in 73^rd^ codon) and *NADPH-dependent L-lysine N(6)-monooxygenase* (*mbtG*, T>A transition at 1238 position in *M. orygis* resulting in Phe→Tyr change in 413rd codon). A mismatch was deliberately incorporated at third nucleotide from the 3’-end of the reverse primers. For *IS1081* gene which is unique and identical for all MTBC species sequence was retrieved from NCBI and primers were designed using Primer3web version 4.1.0 (https://primer3.ut.ee/). All oligonucleotide primers used in this study were synthesized by Integrated DNA Technologies (IDT). Table 2 shows primer sequences of three genes used in this study.

### 2.3 DNA extraction, multiplex PCR optimization and sequencing

DNA samples were extracted from the Mycobacterial organisms with Qiagen bacterial DNA isolation kit (Qiagen, Hilden, Germany) as per manufacturer’s protocol. The multiplex PCR was designed to alter one reaction parameter while maintaining stability in other parameters. Different annealing temperatures (60.7°C-70°C), final concentration of three primer pairs (10 pmol/L to 10 μmol/L) and PCR extension time (30 sec to 40 sec) were standardized to establish the multiplex PCR system using EmeraldAmp Max PCR Master Mix (Takara Bio Inc, Shiga, Japan). The optimized multiplex PCR assay was able to differentiate *M. orygis* from other MTBC based on the product size. PCR products were electrophoresed in 2.5 % agarose for 1 h for and stained with ethidium bromide for visualization using a Gel Doc^™^ EZ Imager Gel Documentation System (Bio-Rad, USA). The product sizes of the amplified fragments were determined by using a 100bp DNA ladder (BR Biochem). The amplified products of *mbtG* and *fadD23* from *M. orygis* were sent for sequencing (Barcode Bioscience, Bangalore) to confirm the presence of SNPs.

### 2.4 Determination of Limit of Detection (LOD)

Genomic DNA from one *M. orygis* isolate was extracted as described earlier. The initial concentration of the genomic DNA was determined. Following that, it was serially diluted through five gradients. Multiplex PCR assay was performed with1 μl of each dilution to evaluate the minimum genomic DNA limit.

### 2.5 Determination of specificity

Genomic DNAs from MTBC isolates like *M. tuberculosis* H37Rv, *M. bovis* AN5, *M. bovis* BCG, *M. tuberculosis* and NTM isolates like *M. fortuitum, M. abscessus, M. chelonae, M. parascrofulaceum, M. novocastrense* and non-Mycobacterium samples like *E. coli*, *S. aureus*, *K. pneumoniae* were used to examine the specificity of the assay.

### 2.6. Application of the multiplex PCR for screening postmortem samples

A total of 85 tuberculous-like lesions in the different tissues of slaughtered cattle like lymph nodes, and other organs, including the lungs, liver, spleen, kidney, peritoneum and pleural cavity were methodically inspected. All the tissues were checked visually and inspected by palpation. A grayish-white or yellowish-white granuloma enclosed in a capsule of variable thickness is typically the hallmark of a tuberculous-like lesion in the organs of cattle. Samples with variable-sized tuberculous-like nodular lesions were aseptically cut (about 2 cm thick), brought to the lab as quickly as possible while keeping the cold chain in place, and stored at −20 °C for downstream experiments. The tissue samples were macerated in a sterile pestle and morter and 25 mg of macerated tissue was taken in the 1.5 ml microcentrifuge tube. DNA was extracted and multiplex PCR was carried out using 1 μl of extracted DNA as stated earlier.

### 2.7 TaqMan PCR for validation of developed multiplex PCR

To validate the result of our in-house developed multiplex PCR assay we tested the positive tissue samples with a multiplex Taqman real-time PCR assay as reported earlier by Halse et al., (2011) and Duffy et al. (2020). This five-probe assay detected presence of RD1, RD9, RD12, Rv0444c and a conserved region external to RD9 (Ext-RD9). Table 3 represents the details of the sequences of primers, probes and labeled reporter dye. This assay was performed in a 20 μl volume using the TaqMan^™^ Multiplex Master Mix (Applied Biosystems, Vilnus, Lithuania). Each reaction mixture was prepared with 2×TaqMan^™^ Multiplex Master Mix, 4 mM MgCl_2_, 450 nM forward and backward primers, 125 nM probes, nuclease-free water, and 1 μl of DNA. Thermal cycling was performed in CFX96 Touch Real-Time PCR Detection System (Bio-Rad, USA). The cycling condition used in this reaction was: 1 cycle at 95 °C for 10 min, followed by 45 cycles at 95 °C for 15 s and 60 °C for 1 min. Manufacturer’s instructions was followed for fluorescence data acquisition, and data analysis. One WGS confirmed *M. orygis* isolate was used as positive control. The target patterns from the RD PCR assay were compared to the signature patterns (Table 4) in order to determine the precise species of MTBC isolates.

## Results

### 3.1 Comparative genome analysis of MTBC genome and primer

**Figure 1:**
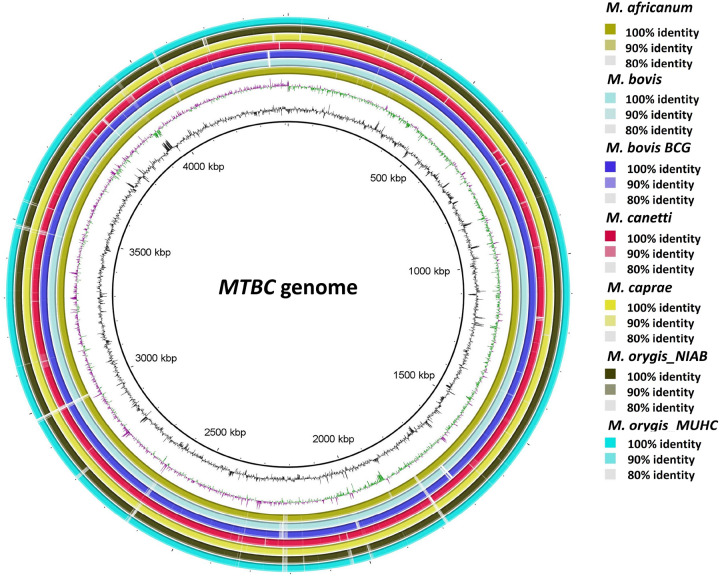
BRIG output image of MTBC member species genome. The innermost rings show GC skew (purple/green) and GC content (black). The reference genome is *M. tuberculosis* H37Rv genome retrieved from NCBI (GenBank accession nos. NC_000962.3). The remaining rings show BLAST comparisons of 6 other complete *MTBC* genomes

**Figure 2:**
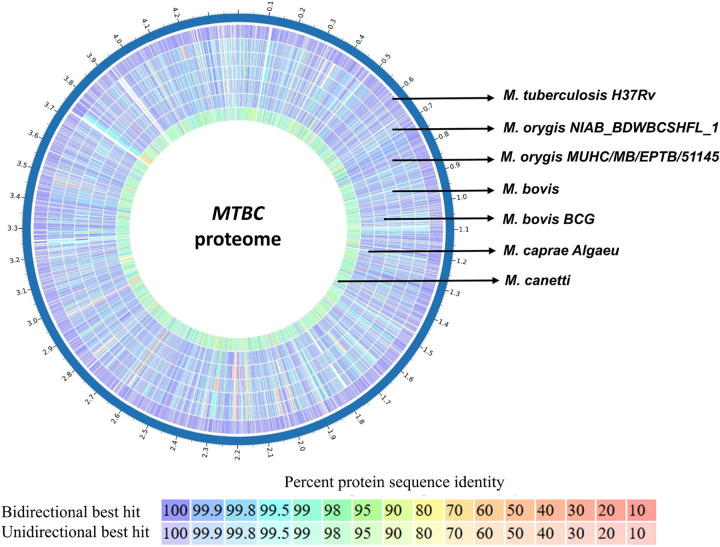
Alignment of the coding regions of the MTBC members. “Proteome Comparison Service” tool of the Bacterial and Viral Bioinformatics Resource Center (BV-BRC) platform was used to align the coding areas of MTBC member species. According to the Best Bidirectional Hits and Unidirectional best hit comparison methods, protein sequence identity is determined on a colorimetric scale, with purple/blue regions denoting a higher percentage of identity than orange/red regions. The white areas indicate absence of coding regions.

**Figure 3:**
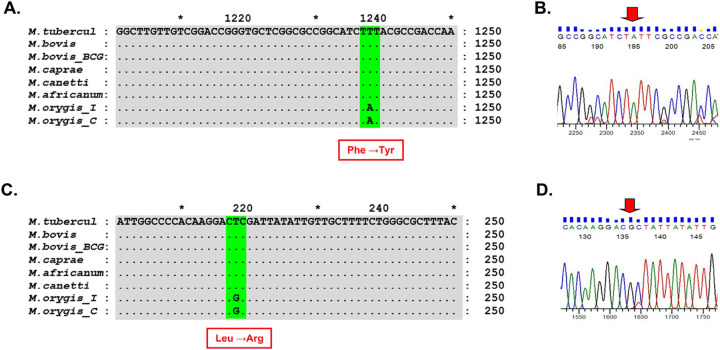
Multiple alignment, PCR and sequencing of amplified products reveal *M. orygis*-specific point mutations in target genes. A) T→A transition at 1238 position of *mbtG* gene of *M. orygis* resulting in change in codon TTT →TAT leading to nonsynonymous change (Phe →Tyr) in primary amino acid sequence of *M. orygis* mbtG protein; B) Sequencing of amplified PCR product confirms the presence of the particular T→A SNP in *M. orygis*(marked with red arrow); C) T→G transversion at 218 position of *fadD23* gene resulting in change in codon CTC →CGC leading to nonsynonymous change (Leu → Arg) in primary amino acid sequence of *M. orygis* fadD23 protein; D) Sequencing of amplified PCR product confirms the presence of the particular T→G SNP in *M. orygis*(marked with red arrow)

BRIG analysis of genome and proteome comparison of different MTBC organisms shows high degree of identity between the species (Fig.1 & 2). So, *M. orygis* specific unique SNPs were targeted for this study. SNP analysis by snippy tools identified a total of 938 SNPs in *M. orygis* genome (Additional data are given in Online Resource 1). Two *M. orygis*-specific SNPs in protein coding genes, and *mbtG* and *fadD23* (Sl. no. 584 and 916 highlighted in blue in Online Resource 1) were randomly chosen for developing the multiplex PCR. The alignment of *mbtG and fadD23* genes across different MTBC species shows two *M. orygis*-specific point mutations viz. T>A transition at 1238 position resulting in Phe>Tyr amino acid change of *mbtG* gene and T>G transversion at 218 position of *fadD23*gene resulting in Leu>Arg amino acid change (Fig. 3 A–D). Accordingly, primers were chosen from the regions of two different genes so that variation in amplicon sizes (240 bp and 181 bp for *mbtG* and *fadD23*, respectively) can be efficiently used for development of multiplex PCR to differentiate *M. orygis* from other MTBC members.

### 3.2 Multiplex PCR Optimization

**Figure 4:**
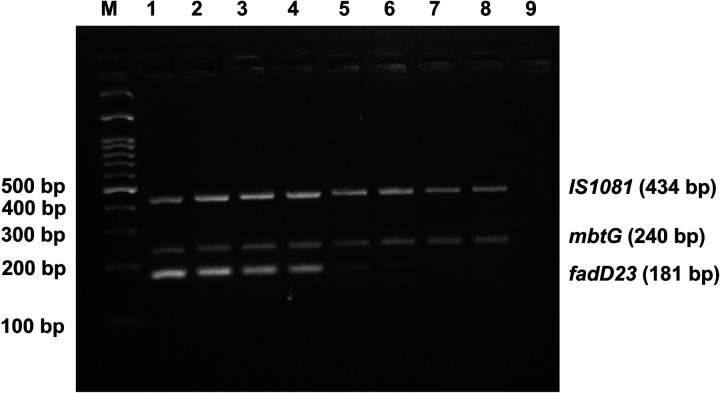
Gradient PCR result for the standardization of annealing temperature. Lane M: 100 bp marker. Lanes 1−8, amplification at different annealing temperatures: 60.7, 61.4, 62.5, 64.2, 66.3, 68.2, 69.3 and 70°C. Lane 9: Non template Control

**Figure 5:**
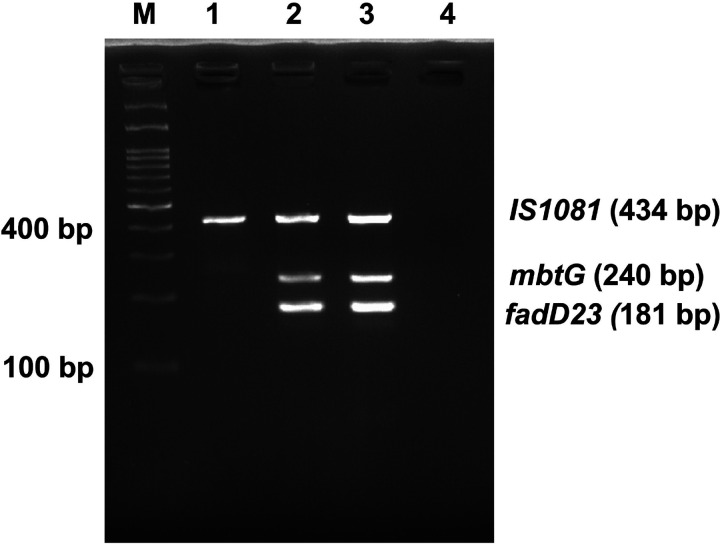
Development of a multiplex PCR assay. M: 100 bp DNA marker. Lane 1: M. tuberculosis H37Rv strain; Lanes 2–3: *M. orygis;* Lane 4: NTC, respectively

The annealing temperature and the ratio of the three primer pairs were standardized. The results showed that IS1081 and two fragments of *mbtG* and *fadD23* genes carrying *M. orygis*-specific mutations were amplified under the PCR condition: 12.5μl of 2 ×EmeraldAmp Max PCR Master Mix, varying volume of six primers and 2 μl of DNA template, 2 μl of deionized water, kept at denaturation at 95 °C for 3 min, 34 cycles of denaturation at 95 °C for 30 s, annealing at 62.5 °C for 30 s, extension at 72 °C for 35 s and final extension at 72 °C for 5 min. A gradient PCR confirmed the optimum amplification of the desired products at an annealing temperature 62.5 °C (Fig. 4). The optimized final primer concentrations used in PCR reaction were 0.12 μM, 0.04μM and 0.4 μM for forward and reverse primers of *IS1081*, *mbtG* and *fadD23*genes respectively. Following electrophoresis in a 2.5 % agarose gel, the amplification products were visualized under UV light. The agarose gel electrophoresis showed the amplified fragments of 240 bp and 181 bp from *M. orygis* only. In addition, 434 bp of IS1081 was amplified from all MTBC strains (Fig. 5). Sequencing chromatogram of the *mbtG* and *fadD23* amplicons confirmed the presence of two specific SNPs in corresponding positions of the two target genes (Fig. 3B and 3D).

### 3.3 Determination of Limit of Detection (LOD)

**Figure 6:**
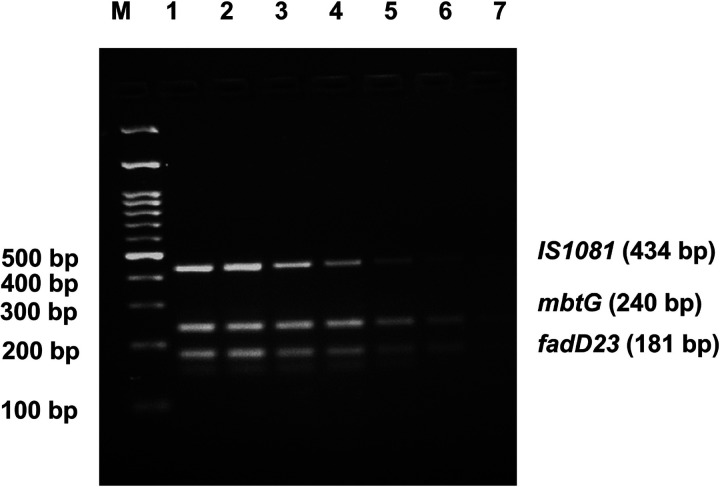
Limit of detection (LOD) of the multiplex PCR assay. M: 100 bp DNA marker. Lane 1: 100 ng DNA; Lanes 2: 20 ng DNA; Lane 3: 4 ng DNA, Lane 4: 0.8 ng DNA, Lane 5: 0.16 ng DNA, Lane 6: 32 pg DNA, Lane 7: 6.4 pg DNA, respectively

The LOD of the developed assay was determined by performing the reactions with 5-fold serial dilution of *M. orygis* DNA quantity ranging from 100 ng to 6.4 pg. As shown in Fig. 6, an LOD of 32 pg *M. orygis* DNA was noted for this multiplex PCR.

### 3.4 Determination of specificity

**Figure 7:**
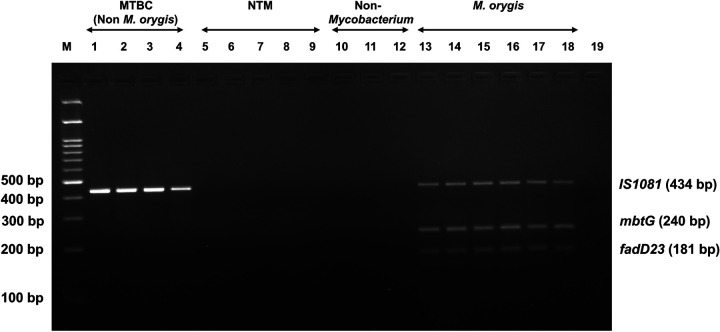
Specificity of the multiplex PCR assay. M: 100 bp DNA marker. Lane 1–4: 434 bp product amplified by *M. tuberculosis* H37Rv, *M. bovis* AN5, *M. bovis BCG and M. tuberculosis* respectively; Lane 5–9: No amplification shown by NTM species like *M. fortuitum*, *M. abscessus, M. chelonae*, *M. parascrofulaceum*, *and M. novocastrense* respectively; Lane 10–12: No amplification shown by non-*Mycobacterium* species like *E. coli, S. aureus, K. pneumoniae*; Lane 13–18: Amplification of *IS1081*, *mbtG*and *fadD23* by six *M. orygis* isolates. Lane 19: No template control

In order to check the specificity of the multiplex PCR assay, we used DNA of *M. tuberculosis* H37Rv, *M. bovis* AN5, *M. bovis* BCG and *M. tuberculosis*, 5 different types of NTM (*M. fortuitum*, *M. abscessus, M. chelonae*, *M. parascrofulaceum*, *and M. novocastrense*), 3 non-*Mycobacterium* (*E. coli*, *S. aureus*, *K. pneumoniae)* and six different isolates of *M. orygis* isolates. The results indicated that primers used to detect the *M. orygis*-specific mutations in two genes yielded 240 bp and 181 bp amplicons specifically and exclusively in the corresponding strains of *M. orygis*. And the primers for MTBC-specific IS1081 generated specific fragments of 434bp for other MTBC members. NTM species and non-*Mycobacterium* species did not show any amplification in the assay (Fig.7). Therefore, the multiplex PCR demonstrated outstanding specificity in detecting strains of *M. orygis*.

### 3.5 Multiplex PCR analysis of postmortem samples

**Figure 8:**
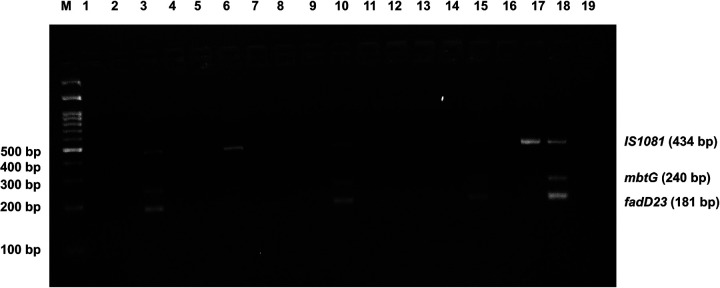
Use of multiplex PCR for screening of tuberculous-like lesions of postmortem tissue samples of cattle. M: 100 bp DNA marker. Lane 3, 10, 15: Samples positive for *M. orygis*; Lane 6: Samples positive for MTBC other than *M. orygis*; Lane 17: Positive control of *M. orygis*; Lane 18: *M. tuberculosis* H37Rv DNA as positive control; Lane 19: No template control

To assess the application of the multiplex PCR assay on identifying the *M. orygis*, 85 tuberculous-like lesions from different organs of slaughtered cattle were used to the multiplex PCR detection assay. As a result, three tissue samples (two lungs and one lymph node) showed bands corresponding to *M. orygis* mutation-specific *mbtG* and *fadD23* genes and one more isolate (lung) showed bands corresponding to MTBC-specific IS1081 gene only (Fig.8). This shows that the in-house developed assay was able to detect *M. orygis* from the suspected tuberculous-like lesions in bovine tissues.

### 3.6 Validation of multiplex PCR with TaqMan Assay

**Figure 9:**
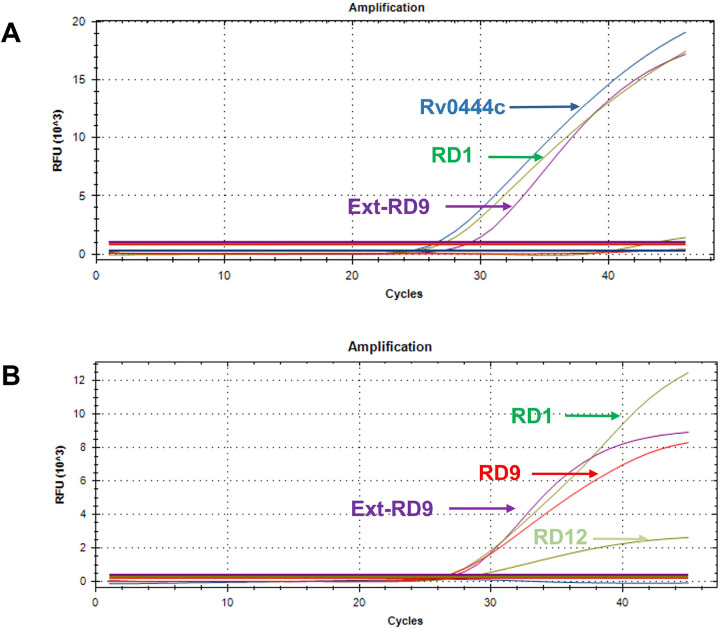
Validation of multiplex PCR assay result by Taqman real-time PCR. (A) Positive amplification of RD1, Ext-RD9 and Rv0444c indicates the presence of *M. orygis*. (B) Positive amplification of RD1, RD4, Ext-RD9 and RD12 indicates the presence of *M. tuberculosis*

A five-probe multiplex TaqMan Real-time PCR assay (Halse et al. 2011 and Duffy et al. 2020) was used to identify species of all five DNA samples which were identified as positive for *M. orygis* and other MTBC in our in-house developed multiplex PCR. All three DNA samples which were identified as of *M. orygis* origin by multiplex PCR matched the *M. orygis*-specific RD-real time PCR pattern listed in Table 4. Another one sample which was identified as MTBC was confirmed as *M. tuberculosis*. Amplification plots of all six DNA samples have been represented in Fig. 9.

## Discussion

*M. orygis* has been reported from 14 countries of 5 different continents, with the exception of those on the South America and Antarctica (data as of March, 2025). 84 out of 250 cases (33.6%) have been recorded from South Asian nations, including Bangladesh, India, Pakistan, and Nepal (Hugh et al. 2025). All these cases are linked to a wide variety of hosts, including several mammalian species including cattle. An extensive molecular epidemiological surveillance study between 2018 and 2019 in India with 940 mycobacterial cultures showed higher prevalence of *M. orygis* than *M. bovis*. This finding also broadened definition of zTB, not limited to *M. bovis* but included other MTBC subspecies like *M. orygis* (Duffy et al. 2020). Apart from human prevalence of this organism has been evidenced in other animal species such as cattle, black buck, spotted deer, and Indian bison (Refaya et al. 2022; Sharma et al. 2023; Haque et al. 2024). The transmission dynamics of *M. orygis* emphasizes the importance of constant attention and improvement of surveillance strategies for effective control of the disease. *M. orygis* and the other MTBC members are closely related at the genome level (Fig.1) and proteome level (Fig.2), making it difficult to distinguish and identify them using conventional methods (Rahim et al. 2007; Islam et al. 2023). Because of diagnostic challenges and underreporting the data regarding actual burden of *M. orygis* and clinical characteristics of *M. orygis* infection is scanty.

The molecular methods of diagnosis of *M. orygis* depend on structural variations in genome. The presence or absence of specific region of difference (RD) regions in genome have been analyzed and it shows the presence of RD1, RD2, RD4, RD5a, RD6, and RD13 and absence of RD7, RD8, RD9, RD10, and RD12 regions in *M. orygis* genome (van Ingen et al. 2012; Refaya et al. 2019). Also, several *M. orygis*-specific SNPs have been identified in earlier studies by whole genome sequencing and comparative genome analysis (Islam et al. 2023; Karthik et al. 2023). However, very few studies are there which have used these genomic markers to detect *M. orygis*. Duffy et al. (2020) developed a Taqman-Real-time PCR which can differentiate *M. orygis* from other MTBC based on specific mutation at Rv0444c. But the use of costly reagents and Taqman probes make this assay expensive to carry out in resource-poor laboratories. In this context, we have developed a simple and low-cost conventional PCR-based multiplex assay for detecting and differentiating *M. orygis* from other MTBC based on two *M. orygis*-specific SNPs.

The increasing number of bacterial genomes that have been sequenced over time has enabled the capturing of genomic variations among various bacterial species. In this study, we analyzed the complete genome of MTBC species to find out SNPs specific for *M. orygis*. We selected only those SNPs which are in protein coding region of DNA for developing this assay. Out of 938 SNPs specific for *M. orygis* 859 SNPs are in coding region of the genome (Online Resource 1). A number of factors that could affect the detection, including the primer sequence, concentration and annealing temperature, have been taken into consideration in order to achieve good specificity, sensitivity, and assay speed. IS1081 specific primers were designed using Primer3 software and in silico PCR (Bikandi et al. 2004) was carried out to check the specificity of the primer. For, primers related to *mbtG* and *fadD23* the mutation point was kept at 3’-end of the reverse primer and single mismatch was deliberately incorporated at third nucleotide from the 3’-end of the reverse primers to increase the specificity (Medrano and De Oliveira 2014). The limit of detection of multiplex PCR assay was 32 pg for *M. orygis* DNA. NCBI-BLAST analysis of the target genes or gene fragment (*IS1081*, *mbtG* and *fadD23*) shows IS1081 is present in five copies while *mbtG* and *fadD23* genes are present in single copies in *M. orygis* genome (Online Resource 2; Fig. 1–3). This indicates that in house developed multiplex PCR is highly sensitive to detect single copy genes from very minute quantity of DNA.

The *mbt* genes in mycobacteria plays role in the biosynthesis of mycobactin, a siderophore crucial for iron uptake and cellular survival of the bacteria. In silico analysis shows that T→A transition at 1238 position of *mbtG* gene of *M. orygis* results in change in codon TTT →TAT leading to nonsynonymous change (Phe →Tyr) in primary amino acid sequence in *M. orygis*. As both Phe and Tyr are aromatic amino acids the change may have limited effect on the functional significance of *M. orygis*-specific *mbtG* gene mutation. Furthermore, Fad23 is a protein involved in the synthesis of Sulfolipid-1, a vital constituent of Mycobacterial cell wall. It belongs to the class of fatty acid adenylating ligases (FAALs), which activate fatty acids for subsequent use in the synthesis of complex lipids and lipopeptides. Yan et al. (2023) recently solved crystal structure of the protein and highlighted its structure-function relationship. The FadD23 N-terminal domain cannot bind palmitic acid on its own without the assistance of the C-terminal domain. Hence it is almost inactive when the C-terminal domain is removed. A nonsynomous mutations (Leu→Arg) has occurred on the structure of Fad23 protein in *M. orygis*. This may have an effect on the functional properties of the protein as both are biochemically different amino acids. A future pathogenomic study should be directed to find out the impact of this change on functional properties of the protein and infectivity of *M. orygis*.

The conventional PCR-based multiplex PCR assay also opens up an opportunity of further investigation whether the assay can be utilized for detection of pathogenic *M. orygis* and its potential role in occupational zoonosis as workers involved in the slaughter of animals and the handling of meats are in at high risk of exposure to this pathogenic MTBC species.

## Conclusion

In conclusion, based on two SNPs in *mbtG* and *faD23* genes the assay developed in this study was shown to be specific for *M. orygis*, which will help with the quick and affordable detection of this zoonotic disease using traditional PCR-based multiplexing. This will be immensely useful for surveillance of *M. orygis* with conventional PCR in resource-poor laboratory set up.

## Supplementary Files

This is a list of supplementary files associated with this preprint. Click to download.

- ESM1.xls
- ESM2.docx
- ESM3.docx

## Figures and Tables

**Table 1:** Details of the bacterial strains used in the study

Bacterial species used	Sources of reference	Host animal	Host tissue/other samples	Year of isolation
*M. orygis* (SRA ID:SRS14280234)	Haque et al. 2024	Cattle	Lung	2019
*M. orygis* (SRA ID:SRS14280233)	Haque et al. 2024	Cattle	Liver	2019
*M. orygis* (SRA ID:SRS5494767)	Our laboratory (WGS submitted on NCBI)	Cattle	Lung	2016
*M. orygis* (SRA ID:SRS14266089)	Our laboratory (WGS submitted on NCBI)	Cattle	Lung	2014
*M. orygis* (SRA ID:SRS5494770)	Our laboratory (WGS submitted on NCBI)	Cattle	Lung	2013
*M. orygis* (SRA ID:SRS5494769)	Our laboratory (WGS submitted on NCBI)	Cattle	Lung	2013
*M. tuberculosis* H37Rv	Reference strain	-	-	-
*M. bovis* AN5	Reference strain	-	-	-
*M. bovis* BCG	Reference strain	-	-	-
*M. tuberculosis*	Our laboratory			
*M. fortuitum*	Haque et al. 2024	Cattle	Liver	2020
*M. abscessus*	Haque et al. 2024	Cattle	Lung	2020
*M. chelonae*	Haque et al. 2024	Cattle	Lung	2020
*M. parascrofulaceum*	Haque et al. 2024	Cattle	Liver	2020
*M. novocastrense*	Haque et al. 2024	Cattle	Lung	2020
*Escherichia coli* (caec9)	Bandyopadhyay et al. 2021	Cattle	Rectal Swab	2020
*Staphylococcus aureus* (VRSA1)	Bhattacharyya et al. 2016	Cattle	Milk	2013
*Klebsiella pneumoniae* (cakp13)	Bandyopadhyay et al. 2021	Cattle	Rectal Swab	2020

**Table 2:** Primer sequences of *IS1081, mbtG*, and *fadD23*

Species	Gene	Primers (5’−3’)	Tm (°C)	Product size (bp)
*All MTBC*	*IS1081*	Forward: AAGGAAATGACGCAATGACC	63	434
		Reverse: CATGATCGACACTTGCGACT	65	
*M. orygis*	*mbtG*	Forward: CTGTTCAGTCAGCACACCCTCG	70	240
		Reverse: GTCGTTGTGTTTGGTCGGCGAAT	71	
	*fadD23*	Forward: ACGGCATTCACTTACATCGATTA	64	181
		Reverse: CCAGAAAAGCAACAATATAATAGC	59	

**Table 3:** Sequences of primers, probes and labeled dye used in Taqman PCR for validation of in house developed multiplex PCR-based identification of MTBC species

Probe/ Primer	Sequence	Dye	Quencher	Reference
Rv0444c_Probe	CTCGGCTGACCCGA	FAM	MGB NFQ	(Duffy et al. 2020)
Rv0444c_Forward	GATGCTGGGCACCATTGTC			
Rv0444c_Reverse	GCCCACCGGTACCATCTTG			
RD1_Probe	CACTCTGAGAGGTTGTCA	VIC	MGB NFQ	(Halse et al. 2011)
RD1_Forward	CCCTTTCTCGTGTTTATACGTTTGA			
RD1_Reverse	GCCATATCGTCCGGAGCTT			
RD9_Probe	AGGTTTCA+CCTTCGAC+CC	TEXAS RED	BHQ	
RD9_Forward	TGCGGGCGGACAACTC			
RD9_Reverse	CACTGCGGTCGGCATTG			
RD12_Probe	TGCGCTGACCCCAC	VIC	MGB NFQ	
RD12_Forward	CGTTGGAACGCGAAATACG			
RD12_Reverse	CCAGGATATGGGCGCAAAT			
EXT-RD9_Probe	G+TT+CTTCAG+CTGGT+CC	CY5	BHQ	
EXT-RD9_Forward	GCCACCACCGACTCATAC			
EXT-RD9_Reverse	CGAGGAGGTCATCCTGCTCTA			

**Table 4:** Amplification profile of Taqman PCR results used to determine MTBC species (Source: Halse et al. 2011 and Duffy et al. 2020)

Organism	RD Target amplification				
	RD1	RD9	RD12	Rv0444c	Ext-RD9
*M. tuberculosis*	**+**	**+**	**+**	**−**	**+**
*M. orygis*	**+**	**−**	**−**	**+**	**+**
*M. bovis*	**+**	**−**	**−**	**−**	**+**
*M. bovis* BCG	**−**	**−**	**−**	**−**	**+**
*M. africanum*	**+**	**−**	**+**	**−**	**+**
*M. microti*	**−**	**−**	**+**	**−**	**+**
NTM	**−**	**−**	**−**	**−**	**−**

## Data Availability

Data will be made available on request.

## References

[R1] Alikhan NF, Petty NK, Ben Zakour NL, Beatson SA (2011) BLAST Ring Image Generator (BRIG): Simple prokaryote genome comparisons. BMC Genomics. 12:402. 10.1186/1471-2164-12-402 21824423 PMC3163573

[R2] Bandyopadhyay S, Bhattacharyya D, Samanta I, Banerjee J, Habib M, Dutta TK, Dutt T (2021) Characterization of Multidrug-Resistant Biofilm-Producing Escherichia coli and Klebsiella pneumoniae in Healthy Cattle and Cattle with Diarrhea. Microbial Drug Resistance 27:1457–1469. 10.1089/MDR.2020.0298 33913759

[R3] Bespiatykh D, Bespyatykh J, Mokrousov I, Shitikov E (2021) A Comprehensive Map of Mycobacterium tuberculosis Complex Regions of Difference. mSphere. 6:10–1128. 10.1128/msphere.00535-21 PMC8386458 34287002

[R4] Bhattacharyya D, Banerjee J, Bandyopadhyay S, Mondal B, Nanda PK, Samanta I, Mahanti A, Das AK, Das G, Dandapat P, Bandyopadhyay S (2016) First report on vancomycin-resistant staphylococcus aureus in bovine and caprine milk. Microbial Drug Resistance 22:675–681. 10.1089/MDR.2015.0330 26990514

[R5] Bikandi J, Millán RS, Rementeria A, Garaizar J (2004) In silico analysis of complete bacterial genomes: PCR, AFLP–PCR and endonuclease restriction. Bioinformatics 20:798–799. 10.1093/BIOINFORMATICS/BTG491 14752001

[R6] Duffy SC, Marais B, Kapur V, Behr MA (2024) Zoonotic tuberculosis in the 21st century. Lancet Infect Dis 24:339–341. 10.1016/S1473-3099(24)00059-8 38307096

[R7] Duffy SC, Srinivasan S, Schilling MA, Stuber T, Danchuk SN, Michael JS, Venkatesan M, Bansal N, Maan S, Jindal N, Chaudhary D, Dandapat P, Katani R, Chothe S, Veerasami M, Robbe-Austerman S, Juleff N, Kapur V, Behr MA (2020) Reconsidering Mycobacterium bovis as a proxy for zoonotic tuberculosis: a molecular epidemiological surveillance study. Lancet Microbe 1:e66–e73. 10.1016/S2666-5247(20)30038-0 32642742 PMC7325494

[R8] Global tuberculosis report 2024. Geneva: World Health Organization; 2020

[R9] Global tuberculosis report 2024. Geneva: World Health Organization; 2024

[R10] Halse TA, Escuyer VE, Musser KA (2011) Evaluation of a single-tube multiplex real-time PCR for differentiation of members of the Mycobacterium tuberculosis complex in clinical specimens. J Clin Microbiol 49:2562–2567. 10.1128/JCM.00467-11 21593269 PMC3147876

[R11] Haque MZ, Guha C, Mukherjee A, Samanta S, Jana PS, Biswas U, Mandal S, Pal S, Venkatesan M, Michael JS, Nanda PK, Bandyopadhyay S, Das AK, Dandapat P (2024) Challenges in diagnosing bovine tuberculosis through surveillance and characterization of Mycobacterium species in slaughtered cattle in Kolkata. BMC Vet Res 20:478. 10.1186/S12917-024-04272-9 39425195 PMC11488179

[R12] Hugh BT, Sim EM, Crighton T, Sintchenko V (2025) Emergence of Mycobacterium orygis: novel insights into zoonotic reservoirs and genomic epidemiology. Front Public Health 13:1568194. 10.3389/FPUBH.2025.1568194 40177079 PMC11961980

[R13] Islam MR, Sharma MK, KhunKhun R, Shandro C, Sekirov I, Tyrrell GJ, Soualhine H (2023) Whole genome sequencing-based identification of human tuberculosis caused by animal-lineage Mycobacterium orygis. J Clin Microbiol 61:e00260–23. 10.1128/JCM.00260-23 37877705 PMC10662373

[R14] Karthik K, Subramanian S, Vinoli Priyadharshini M, Jawahar A, Anbazhagan S, Kathiravan RS, Thomas P, Babu RPA, Gopalan Tirumurugaan K, Raj GD (2023) Whole genome sequencing and comparative genomics of Mycobacterium orygis isolated from different animal hosts to identify specific diagnostic markers. Front Cell Infect Microbiol 13:1302393. 10.3389/FCIMB.2023.1302393 38188626 PMC10770871

[R15] Lipworth S, Jajou R, De Neeling A, Bradley P, Van Der Hoek W, Maphalala G, Bonnet M, Sanchez-Padilla E, Diel R, Niemann S, Iqbal Z, Smith G, Peto T, Crook D, Walker T, Van Soolingen D (2019) SNP-IT Tool for Identifying Subspecies and Associated Lineages of Mycobacterium tuberculosis Complex. Emerg Infect Dis 25:482. 10.3201/EID2503.180894 30789126 PMC6390766

[R16] Medrano RFV, De Oliveira CA (2014) Guidelines for the tetra-primer ARMS-PCR technique development. Mol Biotechnol 56:599–608. 10.1007/S12033-014-9734-4 24519268

[R17] Napier G, Campino S, Merid Y, Abebe M, Woldeamanuel Y, Aseffa A, Hibberd ML, Phelan J, Clark TG (2020) Robust barcoding and identification of Mycobacterium tuberculosis lineages for epidemiological and clinical studies. Genome Med 12:1–10. 10.1186/S13073-020-00817-3/FIGURES/2 PMC7734807 33317631

[R18] Olson RD, Assaf R, Brettin T, Conrad N, Cucinell C, Davis JJ, Dempsey DM, Dickerman A, Dietrich EM, Kenyon RW, Kuscuoglu M, Lefkowitz EJ, Lu J, Machi D, Macken C, Mao C, Niewiadomska A, Nguyen M, Olsen GJ, Overbeek JC, Parrello B, Parrello V, Porter JS, Pusch GD, Shukla M, Singh I, Stewart L, Tan G, Thomas C, VanOeffelen M, Vonstein V, Wallace ZS, Warren AS, Wattam AR, Xia F, Yoo H, Zhang Y, Zmasek CM, Scheuermann RH, Stevens RL (2023) Introducing the Bacterial and Viral Bioinformatics Resource Center (BV-BRC): a resource combining PATRIC, IRD and ViPR. Nucleic Acids Res 51:D678–D689. 10.1093/NAR/GKAC1003 36350631 PMC9825582

[R19] Rahim Z, Möllers M, te Koppele-Vije A, de Beer J, Zaman K, Matin M, Kamal M, Raquib R, van Soolingen D, Baqi M, Heilmann FG, van der Zanden AG (2007) Characterization of Mycobacterium africanum subtype I among cows in a dairy farm in Bangladesh using spoligotyping. Southeast Asian journal of tropical medicine and public health 38:706–713 17883011

[R20] Rani I, Kumar R, Singha H, Riyesh T, Vaid RK, Bhattacharya TK, Shanmugasundaram K (2025) Mycobacterium orygis and its unseen impact: re-evaluating zoonotic tuberculosis in animal and human populations. Front Public Health 13:1505967. 10.3389/FPUBH.2025.1505967 40190750 PMC11968686

[R21] Refaya AK, Kumar N, Raj D, Veerasamy M, Balaji S, Shanmugam S, Rajendran A, Tripathy SP, Swaminathan S, Peacock SJ, Palaniyandi K (2019) Whole-Genome Sequencing of a Mycobacterium orygis Strain Isolated from Cattle in Chennai, India. Microbiol Resour Announc 8:10–1128. 10.1128/MRA.01080-19 PMC6776786 31582446

[R22] Refaya AK, Ramanujam H, Ramalingam M, Rao GVS, Ravikumar D, Sangamithrai D, Shanmugam S, Palaniyandi K (2022) Tuberculosis caused by Mycobacterium orygis in wild ungulates in Chennai, South India. Transbound Emerg Dis 69:e3327–e3333. 10.1111/TBED.14613 35678472

[R23] Rufai SB, McIntosh F, Poojary I, Chothe S, Sebastian A, Albert I, Praul C, Venkatesan M, Mahata G, Maity H, Dandapat P, Michael JS, Katani R, Kapur V, Behr MA (2021) Complete Genome Sequence of Mycobacterium orygis Strain 51145. Microbiol Resour Announc 10: 10–1128. 10.1128/MRA.01279-20 PMC8407732 33414309

[R24] Seeman T. 2015. Snippy: rapid haploid variant calling and core SNP phylogeny. https://github.com/tseemann/snippy.

[R25] Sharma M, Mathesh K, Dandapat P, Mariappan AK, Kumar R, Kumari S, Kapur V, Maan S, Jindal N, Bansal N, Kadiwar R, Kumar A, Gupta N, Pawde AM, Sharma AK (2023) Emergence of Mycobacterium orygis–Associated Tuberculosis in Wild Ruminants, India. Emerg Infect Dis 29:661. 10.3201/EID2903.221228 36823735 PMC9973683

[R26] Soolingen van D, de Haas PEW, Hermans PWM, van Embden JDA(1994) [15] DNA Fingerprinting of mycobacterium tuberculosis. Methods Enzymol 235:196–205. 10.1016/0076-6879(94)35141-4 8057895

[R27] van Ingen J, Rahim Z, Mulder A, Boeree MJ, Simeone R, Brosch R, van Soolingen D (2012) Characterization of Mycobacterium orygis as M. tuberculosis Complex Subspecies. Emerg Infect Dis 18:653. 10.3201/EID1804.110888 22469053 PMC3309669

[R28] World Health Organization. Global tuberculosis report 2020. Geneva: World Health Organization; (2020). https://www.who.int/publications/i/item/9789240013131

[R29] World Health Organization. Global tuberculosis report 2023. Geneva: World Health Organization; (2023). https://www.who.int/publications/i/item/9789240083851

[R30] Yan M, Cao L, Zhao L, Zhou W, Liu X, Zhang W, Rao Z (2023) The Key Roles of Mycobacterium tuberculosis FadD23 C-terminal Domain in Catalytic Mechanisms. Front Microbiol 14:1090534. 10.3389/FMICB.2023.1090534 36896429 PMC9989471

